# Early Identification of Herbicide Stress in Soybean (*Glycine max* (L.) Merr.) Using Chlorophyll Fluorescence Imaging Technology

**DOI:** 10.3390/s18010021

**Published:** 2017-12-22

**Authors:** Hui Li, Pei Wang, Jonas Felix Weber, Roland Gerhards

**Affiliations:** 1 College of Engineering and Technology, Southwest University, Chongqing 400716, China; leehui@swu.edu.cn; 2 Institute of Phytomedicine, University of Hohenheim, 70599 Stuttgart, Germany; j.weber@uni-hohenheim.de (J.F.W.); roland.gerhards@uni-hohenhiem.de (R.G.)

**Keywords:** herbicide stress, phytotoxicity, soybean, chlorophyll fluorescence imaging

## Abstract

Herbicides may damage soybean in conventional production systems. Chlorophyll fluorescence imaging technology has been applied to identify herbicide stress in weed species a few days after application. In this study, greenhouse experiments followed by field experiments at five sites were conducted to investigate if the chlorophyll fluorescence imaging is capable of identifying herbicide stress in soybean shortly after application. Measurements were carried out from emergence until the three-to-four-leaf stage of the soybean plants. Results showed that maximal photosystem II (PS II) quantum yield and shoot dry biomass was significantly reduced in soybean by herbicides compared to the untreated control plants. The stress of PS II inhibiting herbicides occurred on the cotyledons of soybean and plants recovered after one week. The stress induced by DOXP synthase-, microtubule assembly-, or cell division-inhibitors was measured from the two-leaf stage until four-leaf stage of soybean. We could demonstrate that the chlorophyll fluorescence imaging technology is capable for detecting herbicide stress in soybean. The system can be applied under both greenhouse and field conditions. This helps farmers to select weed control strategies with less phytotoxicity in soybean and avoid yield losses due to herbicide stress.

## 1. Introduction

Soybean (*Glycine max* (L.) Merr.) is a worldwide cultivated crop. More than 80% of overall soybeans production originates from the USA, Brazil, and Argentina [1]. Since 1996, the Roundup-Ready (RR) Soybean cultivars have been introduced in the USA, Brazil, and Argentina. Farmers can apply glyphosate as a simple, selective, and effective method for weed control without being concerned about crop injury. In the European Union, weed control in soybean is only performed with conventional herbicides and non-chemical methods. For example, the production of soybean has increased more than 10 times in Germany since 2009 [1]. Pre- and post-emergent herbicide applications are a conventional and effective approach for weed control in soybean cultivations. Occasionally, the herbicides can also damage the crops, delay crop growth, and reduce crop yield when applied under unfavorable soil conditions, weather conditions such as rainfalls and low temperature, or with incorrect timing or mixture [2,3]. Early identification of herbicide stress can contribute to testing the soybean’s genotype sensitivity. It can also help to test the management practices, soil, and weather conditions in order to minimize crop damage, and adjust herbicide dose or select proper herbicide for specific conditions.

Conventional estimation of herbicide damage on crops is usually conducted by visual assessments [4]. For instance, the soybean yield loss can be correlated with the injury symptoms of the stressed plants [5,6]. Advances in computer and photography technology enabled a quantitative assessment method by measuring crop ground cover [4]. A linear relationship was presented between the relative soybean yield and percentage of ground cover. The light reflectance is also used to evaluate the herbicide injury to herbicide [7]. However, these methods evaluate the crop healthiness according to the visible features. That usually requires a relatively long period of time so that the phytotoxic symptoms can be identified on the plants or the plants can grow large enough for the distinction of the ground cover rates. Chlorophyll fluorescence imaging technology is a non-destructive method to investigate the physiological reaction of the photosystem II (PS II) of plants. The approach of chlorophyll fluorescence imaging is very sensitive to abiotic and biotic stress detection on plants [8,9,10]. Some laboratory and greenhouse research demonstrated that, after herbicide application, the chlorophyll fluorescence quantum yield of sensitive weeds was markedly higher than the resistant populations [11,12,13,14,15,16,17]. Wang, Peteinatos, Li, and Gerhards [18] successfully practiced this technology in fields for a survey for resistance profiles of 40 *Alopecurus myosuroides* populations. By applying the chlorophyll fluorescence imaging technology, herbicide efficacy on weeds was observed within five days in the above research. However, the studies and measurements were carried out to distinct herbicide injured sensitive weeds from unstressed resistant population. In that case, herbicide would damage the photosystem II of sensitive weeds. Thus, the variation of chlorophyll fluorescence response can be significant. Recovery of herbicide stress in crops has not been investigated. 

The objective of this study was to test if the herbicide stress and the respective recovery can be identified on the photosystem II of the soybean plants. The question was whether or not this identification can be performed shortly after the herbicide application, and if so what was the time relation between the induction and the identification of the stress. Furthermore, can this identification be performed both under greenhouse and field conditions using the chlorophyll fluorescence imaging technology.

## 2. Materials and Methods

### 2.1. Experimental Design

#### 2.1.1. Greenhouse Experiment

A greenhouse experiment was conducted in the University of Hohenheim (Stuttgart, Germany) from November 2013 until April 2014. Soybeans (Sultana, R.A.G.T. Saaten, Herford, Germany) were sown in pots (15 × 15 × 15 cm) filled with 6.5 kg soil mixture of 50% clay, 25% silt, and 25% sand. The depth of the soil mixture was about 80 mm. The soybeans were sown in a depth of 45 mm with three seeds per pot (equivalent to 96 seeds m^−2^). Plants were grown in a light cycle of 16 h day and 8 h night. The temperature was kept at 25 °C during the day and 15 °C at night. All pots were placed in a complete randomized block design with four blocks. The following three herbicide combinations with recommended dosages were selected for the treatments:

(i) 0.3 kg ha^−1^ Sencor^®^ WG (700 g a.i. kg^−1^ metribuzin, WG, Bayer CropScience) + 0.25 L ha^−1^ Centium^®^ 36 CS (360 g a.i. L^−1^ clomazone, CS, Cheminova Deutschland GmbH) + 0.8 L ha^−1^ Spectrum^®^ (720 g a.i. L^−1^ dimethenamid-P, EC, BASF);
(ii) 2.0 kg ha^−1^ Artist^®^ (175 g a.i. kg^−1^ metribuzin, 240 g a.i. kg^−1^ flufenacet, WG, Bayer CropScience), Harmony^®^ SX^®^ (500 g a.i. kg^−1^ thifensulfuron, SG, Du Pont);
(iii) Harmony^®^ SX^®^ (500 g a.i. kg^−1^ thifensulfuron, SG, Du Pont) + Basagran^®^ (480 g a.i. L^−1^ bentazon, SL, BASF), Harmony^®^ SX^®^ (500 g a.i. kg^−1^ thifensulfuron, SG, Du Pont) + Fusilade^®^ MAX (125 g a.i. L^−1^ fluazifop-P-butyl, EC, Syngenta).

Additionally, for the above herbicide combinations, applications with the half recommended dosages were also sprayed as separate treatments. Untreated control pots with and without hand weeding were included respectively in each block. Herbicide treatments were performed pre- and post-emergence depending on the registrations of the products. The application time is given in Table 1. A laboratory track sprayer chamber mounted with a single flat fan nozzle was used for herbicide application (8002 EVS, TeeJet Spraying System Co., Wheaton, IL, USA). The sprayer was calibrated for an applying volume of 200 L ha^−1^. The applications were performed 500 mm above the soil surface.

#### 2.1.2. Field Experiment

Five field experiments were conducted in 2015. The field trials were located in Southwest Germany at Böblingen, Calw, Nürtingen, Renningen, and Tübingen. All the herbicide combinations were selected according to the local practice of the farmers during the last three years. Seeds of soybeans (Sultana, R.A.G.T. Saaten, Herford, Germany) were sown at a depth of 45 mm between 14 April and 15 May. Approximately 70 seeds m^−2^ were sown with row distance of 170 mm in the fields. The experiments were set up as a randomized complete block design with four blocks and five treatments per block. The size of each plot was 2 × 5 m. The herbicide application was carried out three days after sowing with the following herbicides per treatment,

(i) 2.0 kg ha^−1^ Artist^®^ (175 g a.i. kg^−1^ metribuzin, 240 g a.i. kg^−1^ flufenacet, WG, Bayer CropScience);
(ii) 1.5 kg ha^−1^ Stomp^®^ Aqua (455 g a.i. L^−1^ pendimethalin, CS, BASF) + 2.0 L ha^−1^ Quantum^®^ (600 g a.i. L^−1^ pethoxamid, EC, Cheminova Deutschland GmbH);
(iii) 0.4 L ha^−1^ Sencor^®^ Liquid (600 g a.i. L^−1^ metribuzin, SC, Bayer CropScience) + 0.25 L ha^−1^ Centium^®^ 36 CS (360 g a.i. L^−1^ clomazone, CS, Cheminova Deutschland GmbH);
(iv) 0.4 L ha^−1^ Sencor^®^ Liquid (600 g a.i. L^−1^ metribuzin, SC, Bayer CropScience) + 0.25 L ha^−1^ Centium^®^ 36 CS (360 g a.i. L^−1^ clomazone, CS, Cheminova Deutschland GmbH) + 0.8 L ha^−1^ Spectrum^®^ (720 g a.i. L^−1^ dimethenamid-P, EC, BASF).

An untreated control was included in each block at all sites. Herbicides were sprayed with an electrically motorized plot boom sprayer with Lechler IDK 120-02 nozzles (Metzingen, Germany). The spraying volume was calibrated to 200 L ha^−1^. No rainfall was recorded within 24 h after treatment.

### 2.2. Chlorophyll Fluorescence Sensor

**Figure 1:**
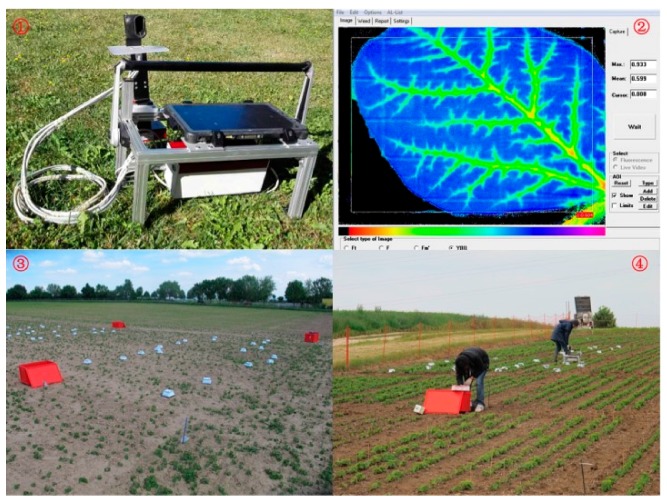
The field chlorophyll fluorescnce sensor WEED-PAM^®^. ① A picture of the sensor. It consists of the camera control unit and the computer including software; ② The software interface when measuring a herbicide treated leaf of soybean. The purple and blue pixels represent leaf area with higher Fv/Fm values, while the red pixels represent leaf area with lower Fv/Fm values. Blue color represents high Fv/Fm values and healthy tissues while the yellow and red color represents pixels with low Fv/Fm values and plant damage; ③ Dark adaption cover box distribution when conducting the first measurement at the one-leaf stage of soybeans at site Böblingen; ④ Measurement at the two-leaf stage of the soybeans at site Nürtingen.

The mobile fluorescence sensor, WEED-PAM^®^ system (Figure 1, Heinz Walz GmbH, Effeltrich, Germany), was used to measure the chlorophyll fluorescence in this research. It contains 40 dark adaption cover boxes, a camera head, a tablet computer, and a central control unit. LED lights emitting light at a wavelength of 460 nm were mounted on the camera head to induce chlorophyll fluorescence. The camera detects fluorescence excitation at above 680 nm after an optical red long pass filter. The efficiency of photosystem II (PS II) of soybeans was determined by measuring the maximal PS II quantum yield (Fv/Fm). It is calculated as

Fv/Fm = (Fm − F_0_)/Fm, (1)

where F_0_ is the minimum fluorescence yield, Fm is the maximal fluorescence yield [8]. The WEED-PAM^®^ system was operated by the software “ImagingWin” (Heinz Walz GmbH, Effeltrich, Germany). With this software, the background noise can be removed as described by Kaiser, Menegat, and Gerhards [16].

### 2.3. Measurements and Data Analysis

For the greenhouse experiment, all measurements with the WEED-PAM^®^ system were conducted 19, 21, 26, 31, 38, and 47 days after sowing (at least one plant had emerged in each pot). One plant per pot was selected for the measurement. All the plants were dark adapted with the dark adaption cover boxes for 30 min before measuring. Whole plants of soybeans were collected and washed 67 days after sowing. The root and aboveground biomass were cut and dried separately. After 48 h drying in a drying chamber under 80 °C, the dry biomass was measured.

For the field trials, three measurements were taken at each site, respectively, when the soybeans were at one-leaf stage (BBCH 10), two-leaf stage (BBCH 11), and three-leaf stage (BBCH 12). Ten soybean plants were measured in each plot. All the plants were dark adapted with the dark adaption cover boxes for 25–30 min before measuring. Values of all 40 plants (four blocks) were averaged. During the measurement, each plant was marked with an orange stick and label, so that the same plants were measured during the experiments. Aboveground biomass was cut on 15 July 2015 (10 to 12 weeks after sowing) at all five sites. Plants were cut in each plot from an area of 0.5 m^2^. The dry aboveground biomass of soybean was weighted after 48 h drying in a drying chamber under 80 °C. 

Data were analyzed with R (Version 3.0.2) and the package agricolae and lawstat [19] (R Development Core Team, 2008). The significance of herbicide effect on soybean plants was determined by performing an ANOVA (*p* > 0.05). In order to separate the treatments, a Tukey’s HSD test (*p* > 0.05) was used. All the datasets were proved to be normally distributed using Shapiro–Wilk test (*p* > 0.05). Homogeneity of variances was analyzed by Levene’s test (*p* > 0.05).

## 3. Results

### 3.1. Greenhouse Experiment

**Figure 2:**
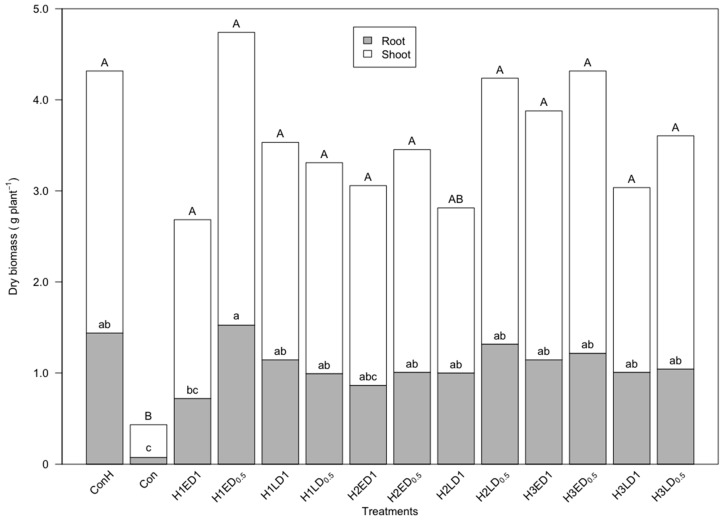
The root and shoot dry biomass per soybean plant on 67 days after sowing. H1, herbicide combination 1; H2, herbicide combination 2; H3, herbicide combination 3; E, early application; L, late application; D1, recommended dosage; D_0.5_, half recommended dosage; significant differences between mean values for the root and the shoot independently are indicated by different letters (Tukey’s HSD Test, *p* < 0.05).

In the greenhouse test, at least one plant emerged in each pot at the performance of the measurements (19 days after sowing). As it can also be seen in Table 2, all three herbicide combinations reduced the Fv/Fm value of the soybean plants (several results were ignored because of overexposure during the measurement). The Fv/Fm of soybeans with pre-emergent herbicide treatments were significantly lower than the control plants during the first three weeks after application. However, the Fv/Fm of plants with post-emergent herbicide application dropped to the lower level only for one week after treatment. Meanwhile, soybeans in treatments with half of the recommended dosage mostly presented no significantly different PS II reaction level than the untreated control plants. Both early and late applications of herbicide led to an Fv/Fm reduction of the soybean plants. Dry biomass measurements (Figure 2) demonstrated that the soybean plants in the untreated group without hand-weeding had the lowest weight. The soybean plants in the untreated group with hand-weeding had relatively high biomass. However, the difference from the herbicide treated groups was not significant.

### 3.2. Field Experiment

The results of the Fv/Fm values and the soybeans’ biomass are presented in Table 3, and their relative change to plants in control groups after herbicide treatment was shown in Table 4.

At Böblingen, the Fv/Fm of soybean seedlings in the treatment i and iv were significantly lower than in the untreated control plants already at the first measurement. However, the plants recovered until the second measurement. The biomass weight of soybean plants with treatment i and iv were significantly lower than the soybean plants of all the other treatments. The biomass of soybean in the plots without herbicide treatment was lowest probably due to weed competition. 

At Calw, the soybean plants presented lower photosystem efficiency in treatment iii. Unlike Böblingen, the herbicide stress on PS II appeared, when plants produced the second leaf. Moreover, the stress lasted until the end of the measurement. Biomass measurements showed the significantly lower weight of soybean in the control and treatment iii than in the other treatments.

A significant response of PS II was observed in treatment ii at Nürtingen. The Fv/Fm values of the soybeans in treatment ii was reduced from the second measuring date until the end of the measurements similar to the trial at Calw. Weed infestation at this site was very low. Therefore, the biomass of soybeans was not reduced in the untreated plots. 

First measurement results of treatment ii and iv at Renningen were lost due to the unexpected power failure when exporting the data from the sensor. At this site, Fv/Fm reduction occurred in treatment iii. However, the difference could only be distinguished until the third leaf of soybeans was produced. The biomass measurements also showed the lower weight of soybeans in the control group and under treatment iii.

At Tübingen, except in the biomass of soybeans in untreated plots, no differences in the PS II quantum yield and the biomass were observed between the treatments.

## 4. Discussion

The chlorophyll fluorescence measurements showed herbicide induced stress on PS II of young soybeans plants in all treatments in the greenhouse, as well as at four sites out of the five field trials. Herbicides with six modes of action were included in the study, which were: PS II inhibition, DOXP synthase inhibition, microtubule assembly inhibition, cell division inhibition, ALS- and ACCase inhibition. Several authors support our findings, that most herbicides reduce light reactions of photosystems shortly after application. Especially when the herbicide dose absorbed by the plants exceeded a certain critical threshold, the plants’ will not be able to metabolize the active ingredients anymore [18,20,21]. 

Metribuzin rapidly inhibits the PS II after treatment by binding at the QB site of plastoquinone and interrupting the electron transfer flow [22]. Most cultivars of soybean are tolerant to metribuzin. Therefore, metribuzin provides selective weed control in soybean [23,24]. Sultana, which was selected for this research, is a metribuzin tolerant cultivator. According to Falb and Smith [25], tolerant soybean cultivators can detoxify metribuzin within 106 hours after treatment. These finding corresponded to our chlorophyll fluorescence imaging measurements revealing a rapid recovery from metribuzin treatments mainly in the field trial at Böblingen. In treatment iii of the greenhouse test, the stress could also be induced by the PS II inhibitor bentazon, as the separated application of thifensulfuron and fluazifop-P-butyl caused no effect on the Fv/Fm of the soybean plants. Biomass assessment showed that post-emergent ALS- and ACCase-inhibiting herbicides did not cause any stress to soybeans. However, their activity against weed species is limited as well. That is why pre-emergent herbicides in soybean production play a major role in weed management.

In the greenhouse study, early occurrence and long duration of stress effect took place after the treatment of herbicide combinations 1 and 2. Apart from the PS II inhibitor-, DOXP synthase-, and cell division- inhibitors were also included in the herbicide mixtures. Thus, another stress mechanism might take place as well in these groups. 

In the field experiments, inhibition of PS II of soybeans at site Calw and Renningen also occurred later and lasted longer than the photosystem regulation at site Böblingen. Besides metribuzin, clomazone (inhibitor of DOXP synthase) was also involved in the stressed treatments. Non-mevalonate 1-deoxy-d-xylulose-5-phosphate (DOXP) pathway is a main biosynthesis approach for plastidic isoprenoids, such as carotenoids, phytol (a side-chain of chlorophylls), plastoquinone-9, isoprene, mono-, and diterpenes [26]. Most of the biosynthesis proceeded inside the chloroplast [27]. Chlorophyll production could be reduced as less phytol was provided due to the DOXP synthase inhibition. Therefore, the photosystem efficiency of DOXP synthase stressed soybeans was lower than the unstressed ones when the plants grew larger. The Fv/Fm reduction of soybean plants in treatment iii at site Calw and Renningen could be attributed to the application of clomazone.

The combined application of pendimethalin (microtubule assembly inhibitor) and pethoxamid (cell division inhibitor) induced stress on PS II at site Nürtingen. Dinitroaniline herbicides like pendimethalin bind to α-tubulin [28]. Thus, the free tubulin could not group into polymerization as microtubule. Several publications noted that dinitroanilines could interfere with the photosystem II dramatically by oxygen evolution [29,30]. Chloroacetamides inhibits the very-long-chain fatty acids (VLCFA) synthase. The herbicide markedly reduces VLCFA content in the plasma membrane and results in cell death [31]. Some chloroacetamides (e.g., carbetamide) could inhibit electron transport up to 50% as a secondary effect of membrane destabilization [21,32]. Therefore, the chlorophyll fluorescence of plants can be altered. This hypothesis correlated well with the Fv/Fm regulation of soybean under the treatment ii combination of metribuzin and flufenacet in the greenhouse test. However, metribuzin was not to be the only compound causing stress in soybean. As the herbicides inhibiting either cell division or VLCFA synthase might induce the regulation on photosystem, the stress mechanism in the treatment ii at site Nürtingen still could not be clearly explained. Furthermore, considering the long period stress on soybeans under the treatment i in the greenhouse experiment, it could also be induced by the combined effect of DOXP synthase- and cell division-inhibitors after the effect of PS II inhibitor metribuzin.

The biomass assessment on herbicide treated soybean significantly distinct the stressed or non-stressed groups in the field. Apparently, the biomass assessment results were similar to the sensor measurements. A similar relationship was also observed in the greenhouse study.

Beside using the Fv/Fm values, several other parameters of chlorophyll fluorescence measurements, such as ΦPSII (effective quantum yield of photochemical energy conversion in PSII) and NPQ (non-photochemical dissipation of absorbed energy), are also common for stress assessment of plants. 

ΦPSII can be measured without dark adaption. However, the measurements require steady-state photosynthesis lighting conditions, which means that plants in the field should be in the full sunlight, and not under any canopy cloudy conditions [8]. As the measurements with Weed-PAM^®^ usually take more than two hours for each site, the weather conditions cannot be ensured for such a long period. Moreover, the system is designed for early herbicide stress detection, the measurements should be conducted within seven days no matter if the weather is sunny, cloudy, or rainy. Thus, spending 20 min for dark adaption and measuring the parameter Fv/Fm should be more appropriate for the sensors field practice.

The other common parameter of chlorophyll fluorescence measurements, NPQ, also can be measured while the plants had a dark adaption period. Yet NPQ is more heavily affected by non-photochemical quenching that reflects heat-dissipation of excitation energy in the antenna system. Thus, it is more often used to indicate the excess radiant energy dissipation to heat in the PSII antenna complexes [33].

WEED-PAM^®^ technology allows quantifying soybean response to herbicide treatments. The variation of plants’ chlorophyll fluorescence emission could be detected shortly after treatment. Thus, herbicide damage to soybean can be avoided by proper selection of products. Since soybean cultivars respond differently to herbicides, WEED-PAM^®^ technology can help to select the most tolerant cultivars. 

## 5. Conclusions

Herbicides interfere directly or indirectly with the photosystem of plants and can reduce quantum use efficiency of PSII in soybean plants and result in lower biomass. With the chlorophyll fluorescence imaging technology, we were capable to identify the herbicide stress rapidly in the young growth stages of the soybean plants. This achievement will help farmers to avoid herbicide combinations that reduces crop growth. Besides, this study showed that the Fv/Fm values of the untreated soybean plants were different at each experiment site. A normalized model should be applied in the further development of the sensor system so that a unified assessment of the stress effect on plants can be created and comparisons can be performed.

## Figures and Tables

**Table 1:** The herbicide application times for the greenhouse experiment (in days after sowing of soybeans). H1, herbicide combination 1; H2, herbicide combination 2; H3, herbicide combination 3; E, early application; L, late application; D_1_, recommended dosage; D_0.5_, half recommended dosage.

Treatments	Days after Sowing						
	Before Emergence		After Emergence				
	4	11	24	31	33	38	45
H1ED_1_	*metribuzin, clomazone, dimethenamid-P*						
H1ED_0.5_							
H1LD_1_		*metribuzin, clomazone, dimethenamid-P*					
H1LD_0.5_							
H2ED_1_	*metribuzin, flufenacet*			*thifensulfuron*			
H2ED_0.5_							
H2LD_1_		*metribuzin, flufenacet*				*thifensulfuron*	
H2LD_0.5_							
H3ED_1_			*thifensulfuron, bentazon*		*thifensulfuron, fluazifop-P-butyl*		
H3ED_0.5_							
H3LD_1_					*thifensulfuron, bentazon*		*thifensulfuron, fluazifop-P-butyl*
H3LD_0.5_							

**Table 2:** The results of chlorophyll fluorescence measurements (Fv/Fm means) of the greenhouse experiment. H1, herbicide combination 1; H2, herbicide combination 2; H3, herbicide combination 3; E, early application; L, late application; D_1_, recommended dosage; D_0.5_, half recommended dosage; ConH, control with hand weeding; Con, control without hand weeding; significant differences between mean values are indicated by different letters (Tukey’s HSD Test, *p* < 0.05).

Treatments	Days after Sowing											
	19		21		26		31		38		47	
H1ED_1_	0.264	b	0.241	cd	0.271	cd	0.484	bc	0.717	a	0.724	a
H1ED_0.5_	0.425	ab	0.520	abc	0.483	abc	0.608	abc	0.739	a	0.731	a
H1LD_1_	0.330	b	0.386	bcd	0.361	bcd	0.605	abc	0.740	a	0.725	a
H1LD_0.5_	0.463	ab	0.466	abcd	0.405	abcd	0.577	abc	0.708	a	0.716	a
H2ED_1_	0.296	b	0.285	cd	0.295	cd	0.476	bc	0.723	a	-	
H2ED_0.5_	0.420	ab	0.419	abcd	0.336	cd	0.515	abc	0.720	a	0.697	a
H2LD_1_	0.235	b	0.201	d	0.152	d	0.432	c	0.720	a	0.705	a
H2LD_0.5_	0.306	b	0.267	cd	0.345	cd	0.567	abc	0.727	a	0.714	a
H3ED_1_	0.655	a	0.695	a	0.425	abcd	0.644	abc	0.737	a	0.724	a
H3ED_0.5_	0.652	a	0.690	a	0.537	abc	0.679	ab	0.746	a	0.729	a
H3LD_1_	0.641	a	0.691	a	0.667	a	0.668	ab	0.666	a	0.705	a
H3LD_0.5_	0.616	a	0.671	ab	0.650	ab	0.673	ab	0.707	a	0.722	a
ConH	0.641	a	0.674	a	0.694	a	0.720	a	-		0.751	a
Con	0.636	a	0.636	ab	0.643	ab	0.672	ab	-		0.733	a

**Table 3:** The results of chlorophyll fluorescence (means of Fv/Fm values) and dry biomass measurements of the field experiment. MoA, Mode of Action; C1, Inhibition of PS II; F4, Inhibition of DOXP synthase; K1, Inhibition of microtubule assembly; K3, Inhibition of cell division (VLCFA); *, stress efficacy indicated by significantly different Fv/Fm values and biomass in both measurements; significant differences between mean values are indicated by different letters (Tukey’s HSD Test, *p* < 0.05).

Sites	Treatment	MoA	Fv/Fm			Biomass (g m^2^)	Stress Efficacy
			Date 1	Date 2	Date 3		
Böblingen	Control	-	0.575a	0.587a	0.666a	310b	
	i	C1 K3	0.423b	0.503a	0.681a	394b	*
	ii	K1 K3	0.543a	0.607a	0.639a	476a	
	iii	C1 F4	0.490ab	0.567a	0.674a	450a	
	iv	C1 F4 K3	0.428b	0.524a	0.639a	356b	*
Calw	Control	-	0.584a	0.558ab	0.672a	40b	
	i	C1 K3	0.575a	0.524bc	0.645ab	296a	
	ii	K1 K3	0.585a	0.571ab	0.647ab	226ab	
	iii	C1 F4	0.596a	0.464c	0.563c	130b	*
	iv	C1 F4 K3	0.585a	0.593a	0.627b	248ab	
Nürtingen	Control	-	0.586a	0.602a	0.722a	580a	
	i	C1 K3	0.629a	0.531ab	0.706a	548a	
	ii	K1 K3	0.586a	0.516b	0.644b	490b	*
	iii	C1 F4	0.583a	0.592a	0.714a	558a	
	iv	C1 F4 K3	0.601a	0.577ab	0.709a	526a	
Renningen	Control	-	0.411a	0.472a	0.645ab	102b	
	i	C1 K3	0.440a	0.513a	0.613b	206a	
	ii	K1 K3	-	0.474a	0.666a	242a	
	iii	C1 F4	0.498a	0.490a	0.426c	136b	*
	iv	C1 F4 K3	-	0.514a	0.632ab	216a	
Tübingen	Control	-	0.545a	0.545a	0.662a	85b	
	i	C1 K3	0.529a	0.478a	0.659a	147a	
	ii	K1 K3	0.555a	0.472a	0.663a	125a	
	iii	C1 F4	0.517a	0.518a	0.658a	150a	
	iv	C1 F4 K3	0.545a	0.520a	0.667a	110a	

**Table 4:** The relative change of the Fv/Fm values and the dry biomass to the untreated control plants of each site and measuring date in the field experiment. The relative Fv/Fm values were calculated on the average Fv/Fm values of the treated plants by the average Fv/Fm values of the relative untreated control plants. MoA, Mode of Action; C1, Inhibition of PS II; F4, Inhibition of DOXP synthase; K1, Inhibition of microtubule assembly; K3, Inhibition of cell division (VLCFA); *, stress efficacy on biomass correlated to significantly different Fv/Fm values in both measurements.

Sites	Treatment	MoA	Relative Fv/Fm			Relative Biomass
			Date 1	Date 2	Date 3	
Böblingen	i	C1 K3	0.736	0.857	1.023	1.271*
	ii	K1 K3	0.944	1.034	0.959	1.535
	iii	C1 F4	0.852	0.966	1.012	1.452
	iv	C1 F4 K3	0.744	0.893	0.959	1.148*
Calw	i	C1 K3	0.985	0.939	0.96	7.4
	ii	K1 K3	1.002	1.023	0.963	5.65
	iii	C1 F4	1.021	0.832	0.838	3.250*
	iv	C1 F4 K3	1.002	1.063	0.933	6.2
Nürtingen	i	C1 K3	1.074	0.882	0.978	0.945
	ii	K1 K3	1	0.857	0.892	0.845*
	iii	C1 F4	0.995	0.983	0.989	0.962
	iv	C1 F4 K3	1.026	0.958	0.982	0.907
Renningen	i	C1 K3	1.071	1.087	0.95	2.02
	ii	K1 K3	-	1.004	1.033	2.373
	iii	C1 F4	1.212	1.038	0.66	1.333*
	iv	C1 F4 K3	-	1.089	0.98	2.118
Tübingen	i	C1 K3	0.971	0.877	0.995	1.729
	ii	K1 K3	1.018	0.866	1.002	1.471
	iii	C1 F4	0.949	0.95	0.994	1.765
	iv	C1 F4 K3	1	0.954	1.008	1.294

## Abbreviation

ANOVA	Analysis of Variance
BBCH	Biologische Bundesanstalt, Bundessortenamt und Chemische Industrie
C1	Inhibition of Photosystem II
CS	Capsule Suspensions
DOXP	1-Deoxy- d-xylulose 5-phosphate
EC	Emulsifiable Concentrates
F4	Inhibition of DOXP Synthase
Fm	Maximal Fluorescence Yield
Fo	Dark Fluorescence Yield
Fv/Fm	Maximal PS II Quantum Yield
HSD	Honest Significant Difference
K1	Inhibition of Microtubule Assembly
K3	Inhibition of Cell Division
LED	Light-Emitting Diode
MoA	Mode of Action
PAM	Pulse Amplitude Modulation
PS II	Photosystem II
QB	a Protein-bound Plastoquinone
SC	Suspension Concentrates
SG	Soluble Granules
SL	Soluble (liquid) Concentrates
VLCFA	Very Long Chain Fatty Acid
WG	Water-Dispersible Granules
